# Long Axial Field-of-View PET for Ultra-Low-Dose Imaging of Non-Hodgkin Lymphoma during Pregnancy

**DOI:** 10.3390/diagnostics13010028

**Published:** 2022-12-22

**Authors:** Joyce van Sluis, Mar Bellido, Andor W. J. M. Glaudemans, Riemer H. J. A. Slart

**Affiliations:** 1 Medical Imaging Center, Department of Nuclear Medicine and Molecular Imaging, University Medical Center Groningen, University of Groningen, Hanzeplein 1, 9713 GZ Groningen, The Netherlands; 2 Department of Hematology, University Medical Center Groningen, University of Groningen, Hanzeplein 1, 9713 GZ Groningen, The Netherlands; 3 Biomedical Photonic Imaging Group, Faculty of Science and Technology, University of Twente, Drienerlolaan 5, 7522 NB Enschede, The Netherlands

**Keywords:** LAFOV PET, ^18^F-FDG, pregnant, follicular lymphoma, image quality

## Abstract

Generally, positron emission tomography imaging is not often performed in the case of pregnant patients. The careful weighing of the risks of radiation exposure to the fetus and benefits for cancer staging and the swift onset of treatment for the mother complicates decision making in clinical practice. In oncology, the most commonly used PET radiotracer is 2-deoxy-2-[fluorine-18] fluoro-D-glucose (^18^F-FDG), a glucose analog which has established roles in the daily routines for, among other applications, initial diagnosis, staging, (radiation) therapy planning, and response monitoring. The introduction of long axial Field-of-View (LAFOV) PET systems allows for PET imaging with a reduced level of injected ^18^F-FDG activity while maintaining the image quality. Here, we discuss the first reported case of a pregnant patient diagnosed with follicular lymphoma using LAFOV PET imaging for the staging and therapy selection. The acquired PET images show diagnostic quality images with clearly distinguishable areas of lymphadenopathy, even with only 34 MBq of injected ^18^F-FDG activity, leading to a considerable decrease in the level of radiation exposure to the fetus.

## 1. Introduction

Follicular lymphoma (FL) is the most common type of low-grade non-Hodgkin lymphoma (NHL) and, representing approximately 35% of all NHLs, the second most commonly diagnosed lymphoma in the United States and Western Europe [1]. FL arises from B-lymphocytes and can therefore be classified as a B-cell lymphoma. Generally, patients with FL present with asymptomatic lymphadenopathy.

Positron Emission Tomography (PET) integrated with Computed Tomography (CT) is a standard of care used in oncology [2,3,4] and many other applications, such as infectious diseases, cardiology, and neurology, providing both metabolic and anatomic information [5]. In oncology, PET/CT is a commonly used and rapidly evolving technique applied in daily clinical practice for, among other applications, initial diagnosis, cancer staging, radiation therapy planning, and response monitoring [4,6,7]. At present, the most commonly used PET radiotracer is 2-deoxy-2-[fluorine-18] fluoro-D-glucose (^18^F-FDG), a glucose analog, for which the accumulation in tissue is proportional to the glucose utilization [6].

The ability to determine the positions of single annihilation events along the line of response (LOR) with precise timing information (time-of-flight (ToF)) is the main strength of state-of-the-art PET systems. With the introduction of ‘digital’ silicon-photomultiplier (SiPM)-based PET technology, the ToF can be improved to reach 210–400 picoseconds (ps), thereby providing images of an improved quality [8]. Moreover, in the case of the ‘analog’ photomultiplier-tube-based systems, the sensitivity was found to be one of the limiting factors affecting both the temporal and spatial image resolution. Long acquisition times had to be used, and image filtering was often applied to reduce the image noise. The improved sensitivity of the state-of-the-art PET systems results in a substantial increase in the counts, i.e., the detected annihilation photons, which results in improved statistics and, thus, a substantially increased signal-to-noise ratio [9]. The latest development in the field of PET system technology was the introduction of long axial Field-of-View (LAFOV) PET systems, which are also equipped with SiPMs, to the commercial market. To date, few selected sites have such an innovative system installed that is (clinically) operational. These systems surround the patient with more detectors in the axial Field-of-View (FOV) direction, which provides two major improvements [10]: (1) a longer axial coverage, resulting in a higher detection efficiency, as more photon pairs are captured, and (2) one bed position that covers a much larger proportion of the patient. Thus, in the same scanning time, a larger part of the body is covered. 

Recently, the LAFOV Biograph Vision Quadra PET/CT system (Siemens Healthineers, Knoxville, TN, USA) [11] was installed at the department of Nuclear Medicine and Molecular Imaging at the University Medical Center Groningen. This system is essentially composed of four interconnected ‘digital’ Biograph Vision PET systems (Siemens Healthineers, Knoxville, TN, USA) [12]. The extended axial FOV of 106 cm results in a substantial increase in the sensitivity, which allows for improved image quality, even in the case of shorter scan durations and/or lower injected activity [13], compared with the conventional PET/CT systems. 

The current case report describes the first case of a pregnant female with FL imaged on a LAFOV PET system. Additional post-processing of the PET data and the subsequent reconstructions revealed that there was potential to administer ultra-low levels of ^18^F-FDG activity in order to minimize fetal radiation exposure, a technique which could be used in the future imaging of pregnant patients.

## 2. Case Presentation

This case involved an obese 28-year-old female patient (height: 179 cm, weight: 115 kg, BMI: 35.9), G1P0. At 13.5 weeks pregnant, she underwent non-invasive prenatal testing (NIPT), which was abnormal. The NIPT revealed an aberrant pattern with multiple chromosomal aberrations in chromosomes 13 and 18. The differential diagnosis included a maternal (hematologic) malignancy, or a trisomy 18 diagnosis of the fetus.

At 16.2 weeks pregnant, the patient underwent a whole-body MRI scan, which revealed extensive lymphadenopathy in the neck, axillary region, mediastinum, abdomen, and inguinal region. Additional findings were hepatosplenomegaly and bone marrow abnormalities, which were most pronounced in the distal femora and proximal tibiae. The bloodwork showed hemoglobin levels of 10.8 g/dL and a non-elevated LDH of 141 U/L. Following diagnostic imaging, a bone marrow biopsy was performed, confirming a follicular lymphoma of grade 1–2 in the bone marrow. 

At the time of presentation, the patient, at 19 weeks pregnant, had no complaints, and after a multidisciplinary discussion, she was referred to the department of Nuclear Medicine and Molecular Imaging for an ^18^F-FDG PET/CT for the further staging of the follicular lymphoma as either transformed or not so as to determine the best chemotherapy treatment. We informed the patient of the risks of radiation but also explained that using our LAFOV PET, we would be able to maintain the radiation levels well below the 100 mGy malformative risk limit defined by the International Commission on Radiological Protection (ICRP) Publication 84 [14].

Prior to intravenous ^18^F-FDG activity injection, the patient was instructed to avoid strenuous exercise, fast for 6 h, and drink 1 L of water. A plasma glucose level of 4.7 mmol/L was measured before the activity administration. The ICRP Publication 84 also states that using smaller administered activities and longer imaging times during pregnancy can reduce the dose absorbed by the fetus. This is stated to be feasible if the patient is not too sick and is able to remain still for the increased duration of the PET acquisition [14]. Hence, the patient received a lowered weight-based (1.5 MBq/kg) injection of 170 MBq ^18^F-FDG activity, followed by a whole-body 15 min listmode PET acquisition for an increased scan duration of 60 min post-injection (in general, 3 MBq/kg is administered, according to the European Association of Nuclear Medicine (EANM) guidelines, for tumor imaging with ^18^F [6], with the associated scan durations, as per the bed position, depending on the scanner type and bed overlap so as to ensure sufficient count statistics). Using a single static bed position measuring 106 cm in axial length (approximately from vertex to mid-thigh), listmode PET emission data were acquired using a maximum ring difference of 85, i.e., a photon acceptance angle of 18 degrees [11]. Following the updated recommended fetal dose estimates for ^18^F-FDG [15] and taking into account the placental crossover values suggested by Benveniste et al. [16], the average dose administered to the fetus per unit activity administered to the mother is 2.2 × 10^−2^. After administering 170 MBq, the average dose administered to the fetus equals a total of 3.74 mGy (please note, the estimate is for 3 months of gestation, whereas at 19 weeks, the estimated average dose to the fetus is lower).

**Figure 1:**
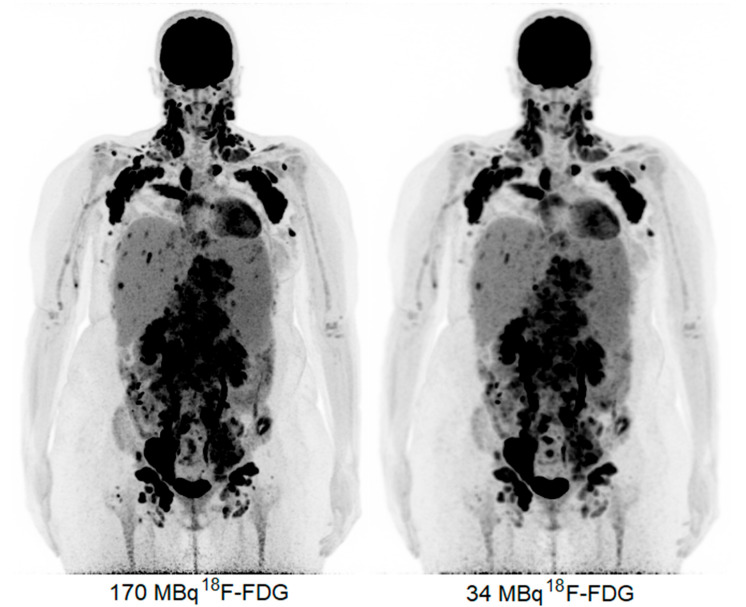
Maximum intensity projection PET images acquired with 170 MBq (**left**) and 34 MBq (**right**).

**Figure 2:**
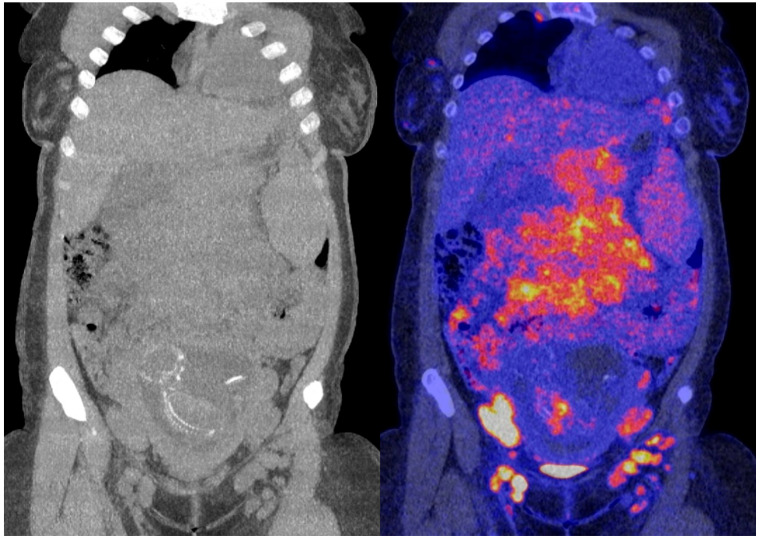
Coronal slice CT (**left**) and fused PET/CT images (**right**) of the fetus.

The obtained ^18^F-FDG PET images show physiologic uptake in the brain, eye muscles, salivary glands, myocardium, liver, spleen, kidneys, and bladder (see Figure 1, left image). Extensive intense pathologic ^18^F-FDG uptake was observed in the lymph nodes above and below the diaphragm (neck bilateral nodal areas, axillae, mediastinum intramammary, mesenteric, retroperitoneal, inguinal, and around the iliac arteries). Other abnormal uptake was observed in the liver and in the musculoskeletal system, e.g., the medial clavicles, vertebral body Th11, the left humeral head, and the left ventral ilium. The disease was staged as stage IV and scored using the follicular lymphoma international prognostic index (FLIPI) with a score of 3 because of the involvement of >4 nodal sites [17]. Figure 2 shows illustrative coronal slice CT and fused PET/CT images of the fetus. 

Subsequently, the PET data were reconstructed and, in addition, the listmode data were reprocessed to simulate acquired images with a lower level of ^18^F-FDG activity administration, e.g., a factor of five times lower, resembling 34 MBq (Figure 1, right image). Here, the average dose administered to the fetus was calculated according to the updated recommended fetal dose estimates, as described above (15), as a result of the 34 MBq ^18^F-FDG activity administered to the mother, and it equaled a total of 0.7 mGy. Images acquired over 15 min were reconstructed using the vendor-recommended, clinically optimized protocol for optimal image reading, consisting of 3D ordinary Poisson (OP-) OSEM with four iterations, five subsets, and a matrix size of 440 × 440 × 708, with a voxel size of 1.6 × 1.6 × 1.5 mm^3^, time-of-flight (ToF) application, and resolution modelling (PSF) without filtering. Additional images obtained resembling the administration of a factor of five times lower ^18^F-FDG activity, e.g., 34 MBq, were reconstructed according to the European Association of Nuclear Medicine Research Ltd. standard 2 [13,18], with the settings consisting of 3D OP-OSEM with four iterations, five subsets, and a matrix size of 220 × 220 × 708, with a voxel size of 3.3 × 3.3 × 1.5 mm^3^, ToF, PSF, and the application of a 5 mm full-width and half-maximum Gaussian filter. These images were considered to be of an adequate image quality to settle the diagnosis of stage IV FL.

Based on the ^18^F-FDG PET presentation, which provided insight into the extent of the disease, combined with earlier FLIPI 3 scoring [17] and an axillary lymph node biopsy, the diagnosis of non-transformed BCL2-break-positive FL was settled, and six cycles of R-CHOP chemotherapy were prescribed. After three cycles, a follow-up ^18^F-FDG PET was performed, which revealed diminished ^18^F-FDG avidity and a lower number of lymph nodes involved with regard to the pre-treatment scan, classified as a partial response. After the fourth cycle, the patient delivered a healthy baby. She is currently finishing the fifth cycle of chemotherapy.

**Figure 3:**
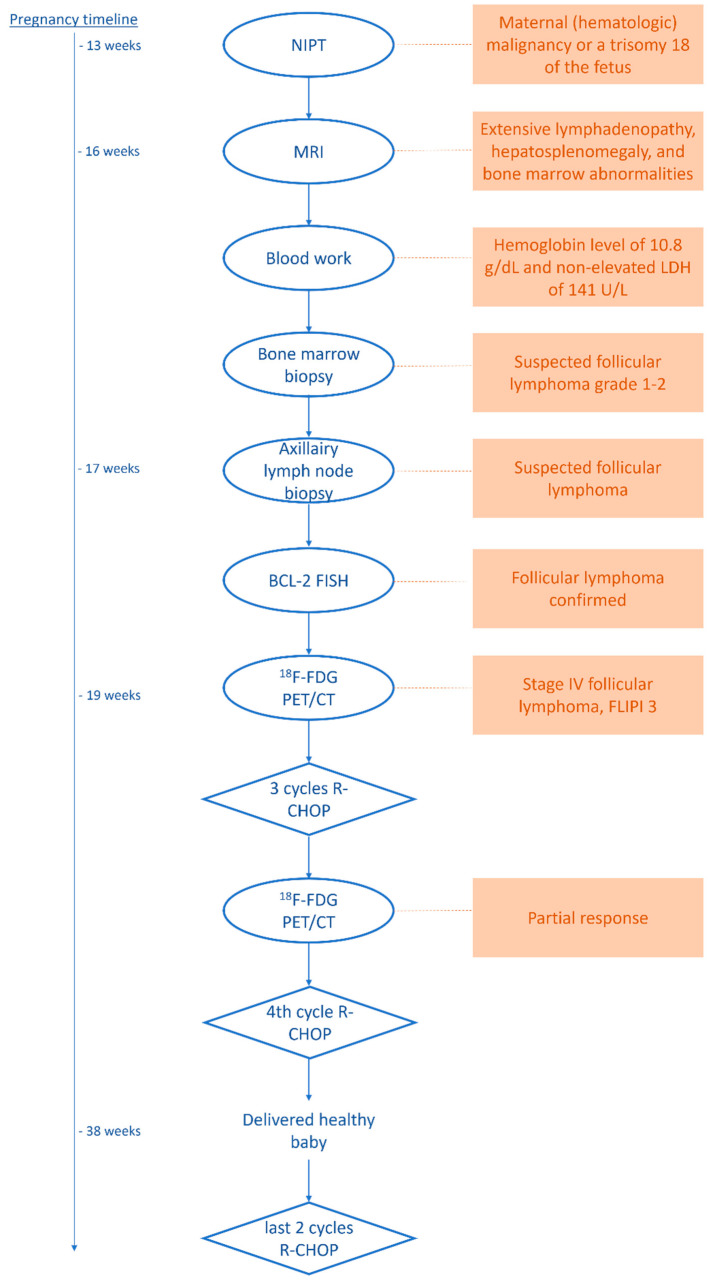
Schematic of the diagnostic procedures, therapy, and follow-up.

For a complete overview of the diagnostic procedures, treatment, and follow-up, please refer to the schematic in Figure 3.

## 3. Discussion

The current case represents a patient population, namely that of pregnant patients, on whom PET imaging is not commonly performed. Weighing the risks and benefits of the increasing number of options of ever-developing and advancing technologies can be challenging, especially when information about their influences on fetal tissues is limited. A previously published case report of interest by Calais et al. (2014) [19] showed the feasibility of performing ^18^F-FDG PET/CT during pregnancy for malignant lymphoma staging, administering the normal administered dose of 3 MBq/kg, according to EANM guidelines. The current case shows that an adequate imaging quality can be achieved using LAFOV PET in pregnant women, even with a 10-fold decrease in the normal dose administered to adults, i.e., instead of administering the recommended 3 MBq/kg, resulting in a 345 MBq ^18^F-FDG injection, in the current presented case, a mere 0.3 MBq/kg dose, resulting in a 34 MBq ^18^F-FDG injection, proved to be sufficient for an adequate image quality. This enables a reduction in the average dose to the fetus from 3.7 to 0.7 mGy. Currently, PET imaging is part of the established daily routine in clinical practice used to detect and stage malignancies. With the emergence of LAFOV PET and its substantially increased sensitivity, it is feasible to lower the radiation exposure to such an extent that even the most vulnerable patient populations can benefit from the potential of PET. 

Alternatively, instead of a significant reduction in the administered activity, for other patient populations, a proportionate reduction in the scan duration can be achieved (or a combination of both). This significant reduction in the scan duration may make it possible to scan other vulnerable patient populations who are unable to lie still for a long time, such as children (without anesthesia) or elderly patients, and patients with, e.g., severe back pain, or claustrophobic patients [9]. In the case of intensive care unit (ICU) patients, on whom PET/CT imaging is currently rarely performed due to logistical issues and the need for continuous monitoring in the case of unstable patients [20], an ultra-fast scan protocol may be beneficial for the use of anatomical CT to obtain one-stop-shop metabolic information, for example, at the possible locations and sources of frequently occurring infections.

Other applications of low-dose or faster PET acquisition procedures can include, for example, the indeterminate pulmonary nodule quantification of ^18^F-FDG uptake to distinguish benign (i.e., inflammatory processes) from malignant diseases as part of a lung cancer screening protocol. In particular, the imaging and quantification of small nodules (<1 cm) [21] at the lung base can be erroneous due to partial volume effects and respiratory motion artifacts [22]. Fast-breath-hold ^18^F-FDG PET, acquiring images within 15–30 s, may be achieved using a LAFOV scanner, which can mitigate these issues [23] and obviate the necessity to apply sophisticated motion correction algorithms [24]. 

Equivalent to imaging methods with less injected radioactive tracer, the increased sensitivity of a LAFOV scanner could be used for delayed imaging with acquisition times post-injection reaching far beyond the possibilities of conventional PET systems, e.g., 2–18 h (10 half-lives) for ^18^F-FDG [25,26]. This prolonged uptake time ensures the increased entrapment of the tracer in the metabolically active tissues. The tumor contrast increases over time and a nearly full washout of the free (i.e., non-metabolized) ^18^F-FDG (background) occurs, resulting in a higher lesion-to-background ratio, i.e., the signal’s specificity increases. Delayed imaging is particularly promising for the detection of metastases in tissues with a high physiological uptake, such as the liver, which decreases over time [27].

Of course, the improved performance characteristics of LAFOV PET can also be used to translate the improved count statistics into an excellent image quality, allowing for smaller voxels while maintaining a high signal-to-noise ratio.

## 4. Conclusions

The first case of ultra-low-dose ^18^F-FDG imaging in oncology during pregnancy using LAFOV PET was described here. This report showed that even with a mere 34 MBq of administered ^18^F-FDG activity (a 10-fold lower administered dose than that defined by the EANM guidelines), a clear image of a more than adequate diagnostic quality can be obtained, limiting the radiation exposure to the fetus substantially compared to the use of conventional PET systems.

## Data Availability

Not applicable.
